# The altered functional status in vestibular migraine: A meta‐analysis

**DOI:** 10.1002/brb3.3591

**Published:** 2024-06-07

**Authors:** Junyong Du, Yong Liu, Wenhao Zhu

**Affiliations:** ^1^ Department of Neurology, Tongji Hospital, Tongji Medical College Huazhong University of Science and Technology Wuhan China; ^2^ School of Artificial Intelligence Beijing University of Posts and Telecommunications Beijing China; ^3^ School of Artificial Intelligence University of Chinese Academy of Sciences Beijing China

**Keywords:** functional connectivity, meta‐analysis, resting‐state fMRI, vestibular migraine

## Abstract

**Purpose:**

Vestibular migraine (VM) is a disorder with prominent vestibular symptoms that are causally correlated with migraine and is the most prevalent neurological cause of episodic vertigo. Nevertheless, the functional underpinnings of VM remain largely unclear. This study aimed to reveal concordant alteration patterns of functional connectivity (FC) in VM patients.

**Methods:**

We searched literature measuring resting‐state FC abnormalities of VM patients in PubMed, Embase, Cochrane, and Scopus databases before May 2023. Furthermore, we applied the anisotropic effect size‐signed differential mapping (AES‐SDM) to conduct a whole‐brain voxel‐wise meta‐analysis to identify the convergence of FC alterations in VM patients.

**Results:**

Nine studies containing 251 VM patients and 257 healthy controls (HCs) were included. Relative to HCs, VM patients showed reduced activity in the left superior temporal gyrus and left midcingulate/paracingulate gyri, and increased activity in the precuneus, right superior parietal gyrus, and right middle frontal gyrus. Jackknife's analysis and subgroup analysis further supported the generalization and robustness of the main results. Furthermore, meta‐regression analyses indicated that the Dizziness Handicap Inventory (DHI) ratings were positively correlated with the activity in the precuneus, while higher Headache Impact Test‐6 and DHI scores were associated with lower activity within the left midcingulate/paracingulate gyri.

**Conclusions:**

The study indicates that VM is associated with specific functional deficits of VM patients in crucial regions involved in the vestibular and pain networks and provides further information on the pathophysiological mechanisms of VM.

## Graphical abstract

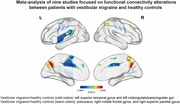

## 1 INTRODUCTION

Vestibular migraine (VM), listed in the appendix of the 3rd edition of the International Classification of Headache Disorders (ICHD‐3) (Headache Classification Committee of the International Headache Society (IHS), 2018), is a disease entity with pronounced vestibular symptoms causally linked to migraine (Marianne Dieterich et al., 2016). VM affects 1% to 3% of the general population and is regarded to be the most prevalent neurological cause of episodic vertigo (M. Dieterich et al., 2016). The possible mechanisms of VM include dysfunction of the trigeminovascular system/nociceptive brainstem centers, aberrant sensory modulation or integration within the thalamo‐cortical network, vestibular system dysfunction, and genetic factors (Huang et al., 2020). Nevertheless, the exact pathophysiology of VM remains largely unknown and the existing explanations for potential mechanisms of VM are mainly in the hypothesis stages, impeding rational management of this prevalent disorder.

Defined as the pattern of synchronous brain activity at rest, resting‐state functional MRI (fMRI)‐based functional connectivity (FC) can describe functional interaction and integration among brain regions and has been proven to be powerful for revealing the pathogenesis of various neuropsychiatric diseases (Biswal et al., 1995; Hutchison et al., 2013; Kenny et al., 2012; Skorobogatykh et al., 2019; Zhu et al., 2019). Several analytical approaches can delineate the features of FC across separate brain areas, such as independent component analysis (ICA)‐based network FC and seed‐based FC (Wu et al., 2018). As a reliable and repeatable approach, seed‐based FC analysis is able to locate the effect regions that are functionally connected to the seed regions directly, while ICA is conducted to parcellate the preprocessed blood oxygen level‐dependent (BOLD) signals into several independent network components and analyze the inter‐ and intranetwork FC.

To date, several studies have found significant FC alterations in multiple brain areas in VM, which were mainly distributed in the anterior cingulate/midcingulate cortex (Chen et al., 2023, 2022; Wang et al., 2023; Zhe et al., 2023), middle frontal gyrus (MFG) (Chen et al., 2023, 2022; Zhe et al., 2021b), precuneus (Chen et al., 2023, 2022), insular cortex (Chen et al., 2023, 2022), supplementary motor area (Chen et al., 2022; Zhe et al., 2021a,b), superior frontal gyrus (Wang et al., 2023; Zhe et al., 2021a), left inferior frontal gyrus (Han et al., 2023), and temporal lobe (Li et al., 2023; Zhe et al., 2021a). However, the results of these studies showed great divergences and even conflicting findings. These heterogeneities may be attributed to the small sample size, the variety in subject recruitment, and the distinct approaches in data processing and analysis among the studies, hindering an improved knowledge of pathophysiological mechanisms in VM. Meta‐analysis of neuroimaging data is an effective strategy to address these challenges, which is capable of synthesizing findings across studies and correlating neuroimaging results with clinical features. However, to date, a quantitative meta‐analysis focused on neural activity alterations in VM has not been carried out yet.

In this study, we applied an anisotropic effect size version of seed‐based d mapping (AES‐SDM), a coordinate‐based meta‐analytic technique with excellent sensitivity and good false positives control, to carry out a whole‐brain voxel‐based meta‐analysis (Radua & Mataix‐Cols, 2012; Radua et al., 2014). The aim of the present study was to assess whether there are any convergent FC alteration patterns that characterize the clinical features in VM patients. The results of this meta‐analysis can provide direct and robust evidence for the mechanisms of functional reorganization and dysregulation in VM.

## 2 METHODS

### 2.1 Literature search

The meta‐analysis was implemented according to the Preferred Reporting Items for Systematic Reviews and Meta‐Analyses (PRISMA) guidelines (Liberati et al., 2009). A literature search was carried out in PubMed, Embase, Cochrane, and Scopus databases to retrieve literature published before May 20, 2023 using the search strategy ‘(“vestibular migraine”) AND (“functional connectivity” OR “network*” OR “graph”) AND (“MRI” OR “BOLD” OR “resting state” OR “rest”)’. In addition, we also searched the references to the included articles and relevant reviews.

### 2.2 Eligible criteria

Original English‐written articles employing resting‐state fMRI were considered when they met the following criteria: (1) recruited right‐handed adult individuals diagnosed with VM based on the ICHD‐3 criteria who were in a symptom‐free phase on the day of MRI scanning; (2) had an age‐ and sex‐matched healthy control (HC) group with no history of migraine, vestibular disorders and chronic pain; (3) case‐control design; (4) reported whole‐brain FC direct comparisons between VM patients and HC participants; (5) provided a detailed description of coordinates in stereotactic space (Talairach or Montreal Neurological Institute [MNI]).

The exclusion criteria were as follows: (1) lacked resting‐state FC method; (2) the method was not based on the whole brain; (3) detailed information from the original article could not be obtained after contacting the authors. In addition, studies on the same samples but analyzed with distinct methods were considered different datasets.

### 2.3 Quality assessment

In consideration of no official guidelines for evaluating the quality of fMRI‐related research, we adopted the criteria as described in a prior study for fMRI studies (Poldrack et al., 2008). Specific grading criteria can be found in Tables S1–S9. A total score of more than 7.5 was judged as good, 4–7.5 as fair, and less than 4 as poor quality. Two authors independently searched the literature, conducted a quality assessment, and extracted imaging data.

### 2.4 Meta‐analysis

This voxel‐wise coordinate‐based meta‐analysis was conducted with the AES‐SDM software version 5.15 (http://www.sdmproject.com/software) (Radua & Mataix‐Cols, 2012; Radua et al., 2012). First, effect size maps were produced using the peak coordinates and statistical values (*p* values or *T* scores, if available) for the clusters of the differences in FC between VM and HC participants for each study. Subsequently, on the same map, positive and negative coordinates were both recreated with the Gaussian Kernel (FWHM = 20 mm). Finally, using a random‐effects meta‐analytic model weighted by sample size, interstudy heterogeneity, and interstudy variance, individual maps from each study were pooled to produce a mean map. In this meta‐analysis, we used the AES‐SDM thresholds that were recommended in a recently published meta‐analysis (voxel *p *< .005, peak height SDM |*Z*| > 1, cluster extent > 100 voxels) (Gao et al., 2019). Based on empirical comparisons, the thresholds were thought to strike a balance between sensitivity and specificity and to be approximately equal to a corrected *p *< .05 in AES‐SDM (Radua et al., 2012).

### 2.5 Reliability analysis

A jackknife sensitivity analysis with the same threshold as the main meta‐analysis was performed to examine the replicability of the results. The analysis was performed repeatedly with removing one study each time. If a finding remains significant in all or the majority of the datasets, it would be considered as robust (Tang et al., 2018). For measuring interstudy variability, a heterogeneity assessment according to Q statistic was employed. In addition, Egger's test was adopted to analyze the probability of publication bias for each significant finding (Egger et al., 1997). A result showing *p *< .05 indicates the presence of publication bias.

### 2.6 Subgroup analysis

A subgroup meta‐analysis of seed‐based FC studies was conducted separately to further explore the potential influence of methodological factors on the main results. The same statistical significance threshold as in the main analysis was adopted.

### 2.7 Meta‐regression analysis

Meta‐regression analyses were implemented to test the potential effects of related demographic and clinical metrics such as mean age, gender proportion, education, disease duration, headache frequency, the Visual Analog Scale (VAS), the Dizziness Handicap Inventory (DHI), and the Headache Impact Test‐6 (HIT‐6). To reduce the identification of erroneous correlations, the thresholds of results were set at an uncorrected value of *p *< .0005 and a cluster extent of 100 voxels.

## 3 RESULTS

### 3.1 Including studies and quality assessment

**FIGURE 1:**
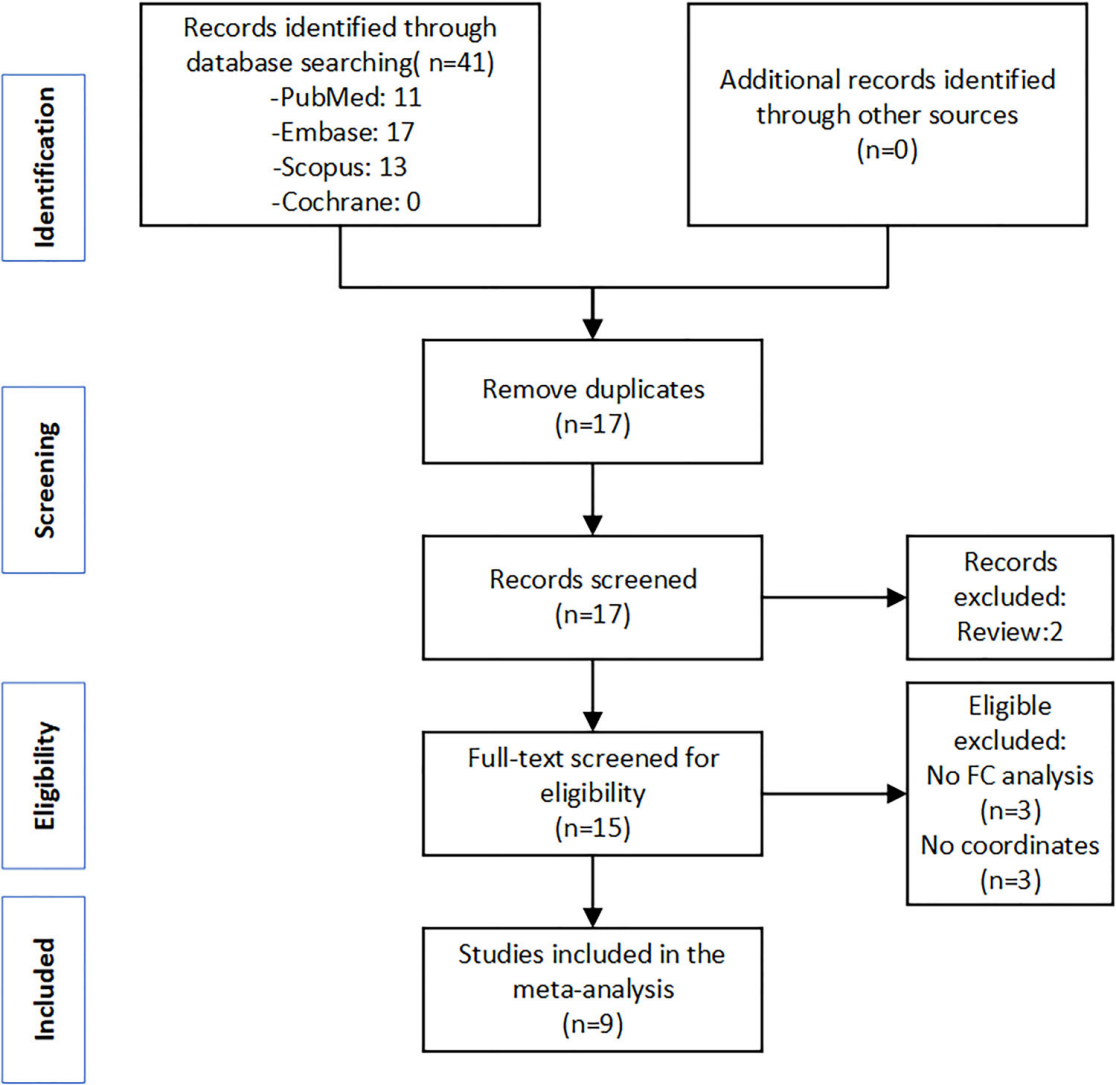
Flowchart depicting the search strategy and retrieved studies. FC, functional connectivity.

As displayed in Figure 1, a total of 41 records were retrieved according to our search strategy (PubMed: 11, Embase: 17, Scopus: 13, Cochrane: 0). During the process of screening, the overlapping datasets that used diverse seeds were considered different datasets. After duplicate removal and literature screening, nine studies (Chen et al., 2023, 2022; Han et al., 2023; Li et al., 2023, 2022; Wang et al., 2023; Zhe et al., 2023; Zhe et al., 2021a,b) were finally eligible for the analysis, comprising 251 VM patients and 257 HCs. Table 1 and Table S10 describe the detailed information of the included studies.

**TABLE 1:** Characteristics of included studies in the meta‐analysis.

Study	Sample size		Female/male (%)		Age (SD), years		Duration (SD), years	Methods	Medication (%)	VAS scores (SD)	HIT‐6 scores (SD)	DHI scores (SD)
	VM	HC	VM	HC	VM	HC						
Wang et al., 2021	18	21	83.3	76.2	36.17 (8.65)	36.15 (12.11)	6.56 (3.36)	Seed based	Drug free	NA	NA	NA
Zhe et al. 2021a	27	25	87.1	86.2	38.22 (10.58)	37.28 (11.45)	9.15 (7.58)	Seed based	29.6%	4.74 (2.75)	51.56 (19.94)	48.93 (16.43)
Zhe et al. 2021b	30	30	90.0	86.7	39.67 (11.10)	37.67 (12.14)	8.39 (7.17)	Seed based	40.0%	5.23 (2.33)	55.93 (12.74)	47.93 (15.07)
Chen et al., 2022	37	44	78.4	63.6	37.81 (7.72)	39.39 (6.84)	6.32 (3.66)	Seed based	59.5%	6.81 (1.76)	56.30 (10.15)	61.32 (19.51)
Han et al. 2023	37	35	89.2	85.7	47.25 (11.05)	43.10 (10.93)	14.1 (12.05)	Seed based	Drug free	6.70 (1.90)	55.50 (10.82)	49.58 (21.37)
Li et al., 2022	17	17	58.8	58.8	39.47 (9.78)	39.82 (13.01)	NA	ICA	NA	NA	NA	NA
Chen et al., 2023	40	40	72.5	62.5	37.92 (7.94)	38.88 (6.59)	6.16 (3.65)	Seed based	NA	6.76 (1.72)	55.77 (10.04)	62.55 (19.08)
Li et al., 2023	17	17	58.8	58.8	39.47 (9.78)	39.82 (13.01)	NA	Seed based	NA	NA	NA	NA
Zhe et al., 2023	28	28	85.7	85.7	40.18 (10.26)	38.25 (12.47)	8.68 (7.52)	ICA	21.4%	5.07 (2.73)	51.86 (19.36)	47.71 (16.04)

DHI, Dizziness Handicap Inventory; HC, healthy controls; HIT‐6, Headache Impact Test‐6; ICA, independent component analysis; NA, not available; SD, standard deviation; VAS, Visual Analog Scale; VM, vestibular migraine.

For study quality evaluation, the score of all included studies yielded a rating of “good” or “fair,” indicating high quality. Three studies were regarded as fair mainly due to the lack of steps of preprocessing and the problem of raw BOLD signals. Tables S1–S9 describe further information about the quality evaluation.

### 3.2 The results of the voxel‐wise meta‐analysis

**FIGURE 2:**
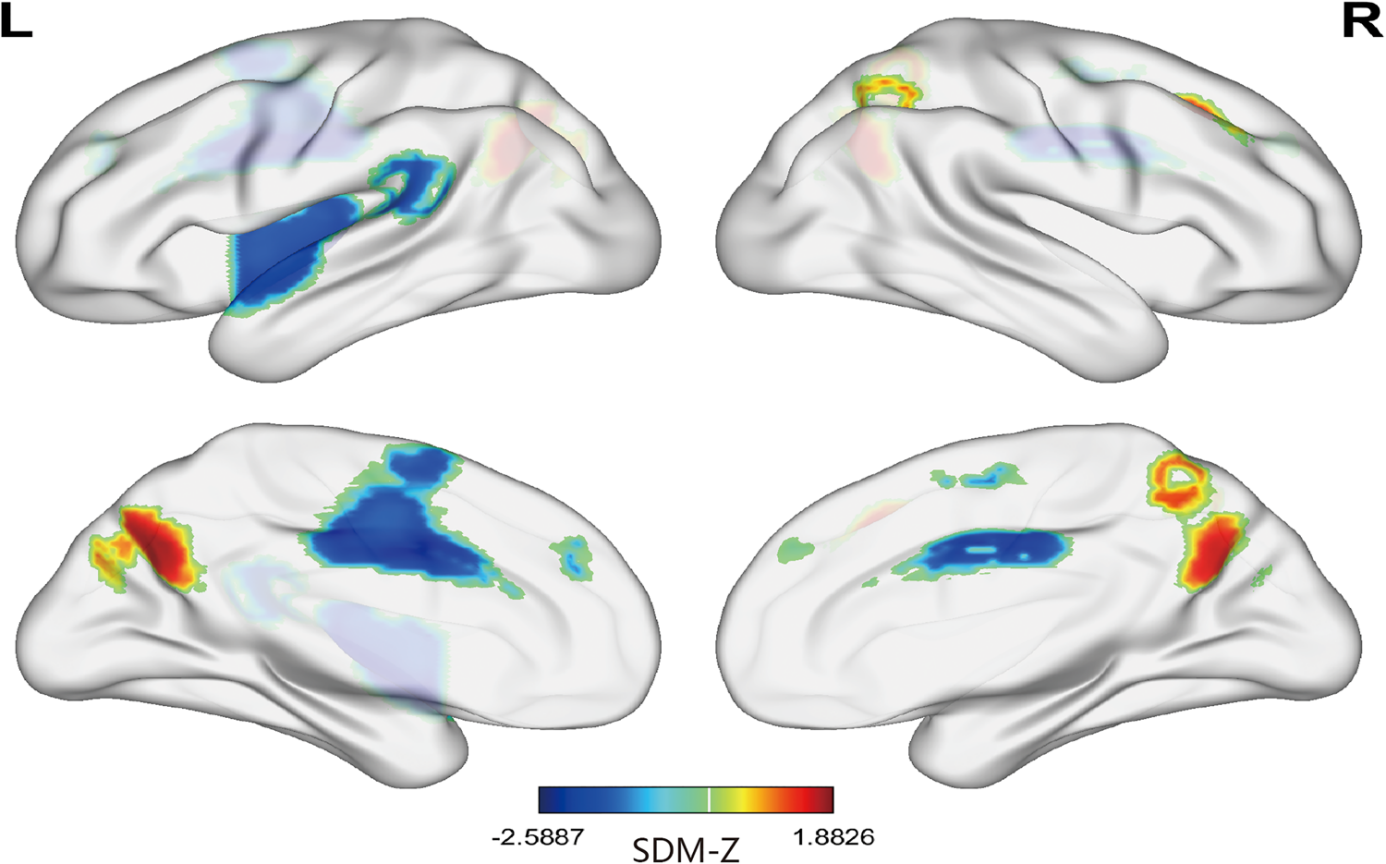
Functional alteration map in vestibular migraine patients compared to healthy controls identified through the voxel‐wise meta‐analysis. Warm colors represent increased activities and cold colors represent reduced activities in VM patients compared to healthy controls. SDM, seed‐based mapping; L, left; R, right.

As shown in Figure 2 and Table 2, compared to HCs, VM patients demonstrated reduced functional activity in the left superior temporal gyrus (STG) (*Z *= −2.385, *p *= .000005) and left midcingulate/paracingulate gyri (*Z *= −2.589, *p *< .000001) while increased activity in the precuneus (*Z *= 1.883, *p *= .000015), right MFG (*Z *= 1.507, *p *= .000377), and right superior parietal gyrus (SPG) (*Z *= 1.375, *p *= .000790).

**TABLE 2:** Clusters with altered functional activity in vestibular migraine patients compared to healthy controls.

Anatomical region	Peak MNI coordinates			SDM‐*Z* value	*p* Value	Cluster size	Jackknife analysis
	*x*	*y*	*z*				
VM > HC							
Precuneus	–8	–66	32	1.883	.000015	1873	4 out of 9
Right middle frontal gyrus	28	28	38	1.507	.000377	252	9 out of 9
Right superior parietal gyrus	26	–58	54	1.375	.000790	169	7 out of 9
VM < HC							
Left superior temporal gyrus	–40	–14	–6	–2.385	.000005	3210	5 out of 9
Left midcingulate/paracingulate gyri	–2	4	34	–2.589	.000001	2443	9 out of 9

HC, healthy control; VM, vestibular migraine.

### 3.3 Reliability analysis

Jackknife sensitivity analysis indicated that the main findings were largely reproducible. The identified clusters in the left midcingulate/paracingulate gyri and right MFG were preserved in all iterations, and the clusters in the right SPG remained significant in all but two dataset combinations. The clusters in the precuneus and left STG remained significant in four and five dataset combinations, respectively. The details are displayed in Table S11.

All identified clusters did not exhibit substantial statistical heterogeneity among studies. Egger's test revealed no publication bias (Figure S1).

### 3.4 Subgroup analysis

The results of subgroup analysis including only seed‐based FC studies were presented in Table S12, among which regions including the precuneus, MFG, and midcingulate/paracingulate gyri were to a large extent in line with the results of the pooled meta‐analysis.

### 3.5 Meta‐regression analyses

**FIGURE 3:**
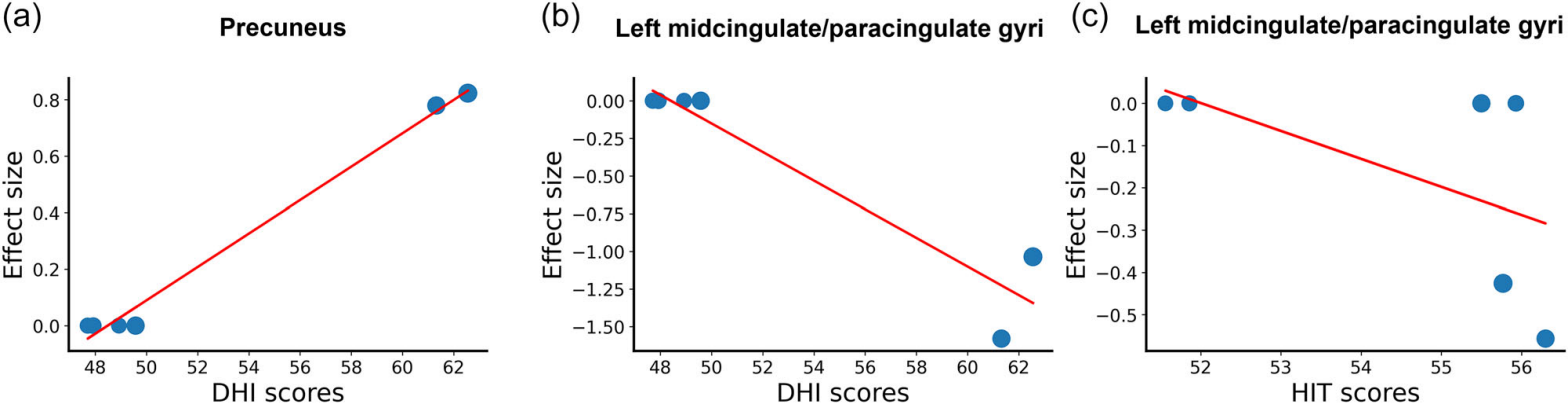
Significant results of meta‐regression analyses. DHI scores were positively correlated with the activity in the precuneus (a), and higher HIT‐6 (b) and DHI scores (c) were associated with lower activity within the left midcingulate/paracingulate gyri. DHI, Dizziness Handicap Inventory; HIT‐6, Headache Impact Test‐6.

The results showed that DHI scores were positively correlated to the activity in the precuneus (*Z *= 3.816, *p *< 1 × 10^−6^). Furthermore, higher HIT‐6 and DHI scores were correlated to lower activity within the left midcingulate/paracingulate gyri (*Z *= −1.359, *p *< 1 × 10^−6^; *Z *= −5.799, *p *< 1 × 10^−6^, respectively) (Figure 3).

## 4 DISCUSSION

The current work is, to our knowledge, the first thorough meta‐analysis to investigate the most consistent and reliable resting‐state FC abnormalities in VM patients. Our results revealed that in contrast to HCs, patients with VM exhibited reduced activity in the left midcingulate/paracingulate gyri and left STG, and increased activity in the precuneus, right MFG, and right SPG. Jackknife's analysis, heterogeneity and publication bias analysis, and subgroup analysis corroborated the primary findings to a great extent. Furthermore, as revealed by meta‐regression analysis, DHI scores were positively correlated with the activity in the precuneus, and higher HIT‐6 and DHI scores were associated with lower activity within the left midcingulate/paracingulate gyri. After diverse results in previous works, our findings provide more robust evidence for elucidating central mechanisms and identifying the crucial aberrant regions in VM.

VM patients exhibited higher activity in the right MFG as compared to HCs. The MFG is considered to be involved in pain perception and regulation (May, 2008) and the dysfunction of the MFG has been demonstrated in patients with migraine (Mengjing Cai et al., 2023). Furthermore, as the origin of fibers that are directly connected to the vestibular nucleus and receive activated vestibular signals (Faugier‐Grimaud & Ventre, 1989), the MFG is also thought to be an important component of the vestibular circuitry (Lobel et al., 1998; Stephan et al., 2005). These findings implicated that the abnormalities in the MFG may be relevant to the development of both vestibular and pain symptoms in VM patients. Interestingly, the subgroup analysis from a recent meta‐analysis of migraine (Zhang et al., 2023) indicated that patients with VM showed a relative structural grey matter (GM) reduction in the right MFG. We thus speculated that the abnormally increased activity in the right MFG may be to some extent explained by a compensatory mechanism. Given that the causal relationship between structural GM and FC alterations remains unknown, this conjecture still needs to be further verified.

We also found increased activity in the precuneus and right SPG of VM patients. The precuneus is a pivotal node of the default mode network (DMN) and is implicated in a variety of higher‐order processes, such as episodic memory, awareness, pain perception, and self‐referential processing, which may be attributed to the unique role of the precuneus on the information transformation and integration across different networks (Cavanna & Trimble, 2006; Dadario & Sughrue, 2023; Mochizuki & Kakigi, 2015). Consistent with our study, a range of pain‐related diseases were shown to have anatomical and functional abnormalities in the precuneus, including migraine (Mengjing Cai et al., 2023; Zhang et al., 2023), diabetic neuralgia (Cauda et al., 2009), fibromyalgia (Napadow et al., 2010), and chronic pain (M. Cai et al., 2023; Malfliet et al., 2019; Zhang et al., 2023), suggesting that the functional aberrant of the precuneus might be linked to the symptom of headache in VM, but not specific. As anatomically adjacent brain regions in the posterior parietal lobe, the precuneus and SPG were both activated in visual motion stimuli and direct attention (Breveglieri et al., 2008; Cavanna & Trimble, 2006; Kikuchi et al., 2009) and were considered to be responsible for the integration of optical and vestibular information (Brandt et al., 1998; Culham et al., 1998; Simon et al., 2002; Wenderoth et al., 2005). Besides, the results of the meta‐regression analysis revealed a close link between functional activity in the precuneus and DHI score for assessing the handicapping effects of dizziness for VM patients, further suggesting that increased FC in the precuneus represents an adaptive response to vestibular symptoms in VM. However, since the precuneus survived in only 4 out of 9 combinations of the datasets in jackknife sensitivity analyses, future researches with a larger sample size are still warranted to verify the speculation.

In the meta‐analysis, we found decreased activity within the midcingulate/paracingulate gyri in VM patients. The midcingulate and paracingulate gyri are demonstrated to be cortical hubs where negative affect, pain, and cognitive control are functionally integrated (Shackman et al., 2011). To be more specific, these regions receive dense fiber projection of the medial thalamic nucleus group and are thought to be key structures in pain processing, perception, and modulation through thalamo‐cortical processing (Tan et al., 2017). Besides, with the connections to the posterior insula, a core node of the parieto‐insular vestibular cortex, the midcingulate cortex is also involved in human vestibular processing (Lopez & Blanke, 2011; zu Eulenburg et al., 2012). Convergent evidence from functional neuroimaging studies suggested that the stimulation of the midcingulate cortex can elicit vestibular responses including vertigo and the feeling of falling into a void (Caruana et al., 2018). Furthermore, as revealed by our study, the HIT‐6 and DHI scores that represent the severity of headache and the handicapping effect of vestibular deficits respectively were both found to be negatively associated with the FC in the midcingulate/paracingulate gyri in VM patients. In addition, the decreased activity within the regions in VM patients was also observed retained in the subgroup analysis of the studies using seed‐based FC analysis separately. Therefore, we could reasonably infer that functional aberrant of the midcingulate/paracingulate gyri may play a critical role in the pathophysiological mechanisms of VM.

The STG is believed to be a fundamental part of the human vestibular network and participates in multisensory integration (Lopez et al., 2012). In line with the evidence of structural changes within the STG in diseases with vestibular symptoms including vestibular neuritis (Helmchen et al., 2009), chronic complete unilateral vestibular deafferentation (Hüfner et al., 2009), and VM (Zhang et al., 2023), the present study identified decreased activity of the STG in VM patients. Hence, it is speculated that the abnormalities in the STG may be partly responsible for the clinical feature of fearful vertigo and hypersensitivity to external sensory stimulation in patients with VM. Nevertheless, the results of decreased activity in the STG were not stable enough in the sensitivity analysis and the subgroup analysis. Therefore, the exact role of the STG in VM needs to be illustrated in the future.

Convergent and divergent clinical characteristics exist in migraine and VM patients. Episodic headache with vestibular abnormalities can be observed in both of the two disorders. Besides, distinct from patients with migraine, the predominant symptom in VM is recurrent severe vertigo attack (Lempert & Neuhauser, 2009), with a higher proportion of motion sickness but lower headache intensity (Özçelik et al., 2022). Our study demonstrated FC alterations in multiple brain regions involving the vestibular processing and migraine circuit, which is in line with vestibular symptoms such as vertigo/dizziness and head‐movement intolerance accompanied by migraine‐like symptoms in VM patients. Furthermore, compatible with the clinical features of the two disorders, VM patients manifested more widespread FC alterations in the brain regions involving the vestibular network than migraine patients, whereas increased activity in the right MFG within the vestibular as well as pain pathway was evident in both of the two disorders (Mengjing Cai et al., 2023; Wen et al., 2023). Altogether, these findings provide further evidence that VM may be considered as a separate entity within the spectrum of migraine‐related disorders that causally connects recurrently prominent vestibular symptoms and migraine attacks (Headache Classification Committee of the International Headache Society (IHS), 2018).

Several limitations in our meta‐analysis should be addressed. First, this meta‐analysis only contains 9 studies with relatively small sample sizes, which may contribute to a limitation of the statistical power that impacted the generalizability of the results. Hence, the meta‐analysis should be considered as an exploratory study, and future research with large samples should be performed to validate the findings. Second, as reported by prior works (Jiang et al., 2020; Wang et al., 2021; Wolters et al., 2019), the results of the research using seed‐based FC analysis and ICA were combined in our analysis to comprehensively investigate FC deficits in VM, which inevitably induced bias due to the different theoretical bases. To overcome this issue, we conducted a subgroup analysis including only seed‐based FC studies, whereas the separate analysis of ICA‐based network FC studies was not able to be implemented on account of the insufficient datasets. Third, heterogeneous clinical conditions and the use of medication may also introduce a risk of bias in the results. However, due to a lack of exact information, it was unable to implement a further subgroup analysis. Finally, VM is the most common cause of vertigo in children and adolescents. Sharing similar diagnostic criteria (van de Berg et al., 2021), VM in children and adolescents also exhibit some distinct clinical characteristics from adults with VM, including more evenly distributed sex ratio (Brodsky et al., 2016; Dieterich & Brandt, 1999; Rosman et al., 2009), higher percentages of family history of migraine (Chen et al., 2022), more often presenting with bilateral migraine headache (Headache Classification Committee of the International Headache Society (IHS), 2018) and less with visual auras (Cokyaman & Cetin, 2022). Nevertheless, it is not able to explore central mechanisms underlying VM in children and adolescents through rsFC analysis since there are insufficient imaging studies focused on these patients.

## 5 CONCLUSIONS

In this study, we performed a whole‐brain voxel‐based meta‐analysis to depict the resting‐state FC alteration patterns in patients with VM. The results demonstrated that the central neural mechanisms of VM represented as multinetwork dysfunction mainly involved in vestibular and pain processing, which provide robust neuroimaging evidence for explaining the core features of prominent vestibular symptoms associated with migraine attacks in VM patients and add impressive support for the existing hypothesis of the possible mechanism of VM. The study contributes to the understanding of the pathophysiological mechanisms underlying VM and provides potential guidance for future investigation.

## AUTHOR CONTRIBUTIONS

**Junyong Du**: Software; data curation; investigation; visualization; validation; writing—original draft; writing—review and editing; formal analysis; project administration; resources. **Yong Liu**: Conceptualization; supervision; writing—review and editing; methodology. **Wenhao Zhu**: Conceptualization; methodology; supervision; funding acquisition; writing—review and editing; project administration; resources; validation; visualization.

## CONFLICT OF INTEREST STATEMENT

The authors declare no conflicts of interest.

### PEER REVIEW

The peer review history for this article is available at https://publons.com/publon/10.1002/brb3.3591.

## Supporting information

Table S1 Wang et al. 2021.
Table S2 Zhe et al. 2021a.
Table S3 Zhe et al. 2021b.
Table S4 Chen et al. 2022.
Table S5 Han et al. 2023.
Table S6 Li et al. 2022.
Table S7 Chen et al. 2023.
Table S8 Li et al. 2023.
Table S9 Zhe et al. 2023.
Table S10 Methodological information of studies.
Table S11 Results of the jackknife sensitivity analysis.
Table S12 Clusters with increased and decreased activity in patients with vestibular migraine compared with healthy controls in the subgroup of seed‐based analysis.
Figure S1 Results of funnel plot analysis to test for publication bias.

## Data Availability

The data that support the findings of this study are available on requests from the corresponding author.
