# Supplementary material for: The altered functional status in vestibular migraine: A meta‐analysis
Source: Brain Behav. 2024 Jun 7;14(6):e3591. doi: 10.1002/brb3.3591 (PMC11161393; doi:10.1002/brb3.3591)

**Supplementary Material for**

**The altered functional status in vestibular migraine: a meta-analysis**

| Supplementary Table S1 Wang et al. 2021 | + | +/- | - | Other (CD, NR, NA)* |
| --- | --- | --- | --- | --- |
| 1. Did they give a full description of the study participants? | V |  |  |  |
| 2. Did they give a full description of the psychological task used in fMRI? | V |  |  |  |
| 3. Did they specify the spatial normalization procedure, including the atlas or template which is used to match the images to? |  |  | V |  |
| 4. Did they specify how the regions of interest were determined? | V |  |  |  |
| 5. Did they provide enough detail to reproduce the analysis? | V |  |  |  |
| 6. Are all the empirical claims supported by a specific statistical test? | V |  |  |  |
| 7. Did they describe and account for the multiple testing problem? | V |  |  |  |
| 8. Do the figures and tables stand on their own? | V |  |  |  |
| 9. Are the quality control measures documented? |  |  | V |  |
| Quality Rating (Good, Fair or Poor) (See guidance) | 7 (fair) | | | |
| Rater #1 initials: | Du | | | |
| Rater #2 initials: | Zhu | | | |
| *Additional Comments (If POOR, please state why):*  *3. The fMRI images are resampled to voxel size 3x3x3mm, while they were obtained with a 3.5-mm slice thickness*  *9.preprocessing lacks removal of linear trend and influence of covariates* | | | | |
| *CD, cannot determine: NA, not applicable: NR, not reported | | | | |

| Supplementary Table S2 Zhe et al. 2021a | + | +/- | - | Other (CD, NR, NA)* |
| --- | --- | --- | --- | --- |
| 1. Did they give a full description of the study participants? | V |  |  |  |
| 2. Did they give a full description of the psychological task used in fMRI? | V |  |  |  |
| 3. Did they specify the spatial normalization procedure, including the atlas or template which is used to match the images to? |  |  | V |  |
| 4. Did they specify how the regions of interest were determined? | V |  |  |  |
| 5. Did they provide enough detail to reproduce the analysis? | V |  |  |  |
| 6. Are all the empirical claims supported by a specific statistical test? | V |  |  |  |
| 7. Did they describe and account for the multiple testing problem? | V |  |  |  |
| 8. Do the figures and tables stand on their own? | V |  |  |  |
| 9. Are the quality control measures documented? |  |  | V |  |
| Quality Rating (Good, Fair or Poor) (See guidance) | 7 (fair) | | | |
| Rater #1 initials: | Du | | | |
| Rater #2 initials: | Zhu | | | |
| *Additional Comments (If POOR, please state why):*  *3. The fMRI images are resampled to voxel size 3x3x3mm, while they were obtained with a 4-mm slice thickness.*  *9. preprocessing lacks removal of linear trend and influence of covariates* | | | | |
| *CD, cannot determine: NA, not applicable: NR, not reported | | | | |

| Supplementary Table S3 Zhe et al. 2021b | + | +/- | - | Other (CD, NR, NA)* |
| --- | --- | --- | --- | --- |
| 1. Did they give a full description of the study participants? | V |  |  |  |
| 2. Did they give a full description of the psychological task used in fMRI? | V |  |  |  |
| 3. Did they specify the spatial normalization procedure, including the atlas or template which is used to match the images to? |  |  | V |  |
| 4. Did they specify how the regions of interest were determined? | V |  |  |  |
| 5. Did they provide enough detail to reproduce the analysis? | V |  |  |  |
| 6. Are all the empirical claims supported by a specific statistical test? | V |  |  |  |
| 7. Did they describe and account for the multiple testing problem? | V |  |  |  |
| 8. Do the figures and tables stand on their own? | V |  |  |  |
| 9. Are the quality control measures documented? | V |  |  |  |
| Quality Rating (Good, Fair or Poor) (See guidance) | 8 (Good) | | | |
| Rater #1 initials: | du | | | |
| Rater #2 initials: | zhu | | | |
| *Additional Comments (If POOR, please state why):*  *3. The fMRI images are resampled to voxel size 3x3x3mm, while they were obtained with a 4-mm slice thickness* | | | | |
| *CD, cannot determine: NA, not applicable: NR, not reported | | | | |

| Supplementary Table S4 Chen et al. 2022 | + | +/- | - | Other (CD, NR, NA)* |
| --- | --- | --- | --- | --- |
| 1. Did they give a full description of the study participants? | V |  |  |  |
| 2. Did they give a full description of the psychological task used in fMRI? | V |  |  |  |
| 3. Did they specify the spatial normalization procedure, including the atlas or template which is used to match the images to? | V |  |  |  |
| 4. Did they specify how the regions of interest were determined? | V |  |  |  |
| 5. Did they provide enough detail to reproduce the analysis? | V |  |  |  |
| 6. Are all the empirical claims supported by a specific statistical test? | V |  |  |  |
| 7. Did they describe and account for the multiple testing problem? | V |  |  |  |
| 8. Do the figures and tables stand on their own? | V |  |  |  |
| 9. Are the quality control measures documented? | V |  |  |  |
| Quality Rating (Good, Fair or Poor) (See guidance) | 9 (Good) | | | |
| Rater #1 initials: | Du | | | |
| Rater #2 initials: | Zhu | | | |
| *Additional Comments (If POOR, please state why):* | | | | |
| *CD, cannot determine: NA, not applicable: NR, not reported | | | | |

| Supplementary Table S5 Han et al. 2022 | + | +/- | - | Other (CD, NR, NA)* |
| --- | --- | --- | --- | --- |
| 1. Did they give a full description of the study participants? | V |  |  |  |
| 2. Did they give a full description of the psychological task used in fMRI? | V |  |  |  |
| 3. Did they specify the spatial normalization procedure, including the atlas or template which is used to match the images to? |  |  | V |  |
| 4. Did they specify how the regions of interest were determined? | V |  |  |  |
| 5. Did they provide enough detail to reproduce the analysis? | V |  |  |  |
| 6. Are all the empirical claims supported by a specific statistical test? | V |  |  |  |
| 7. Did they describe and account for the multiple testing problem? | V |  |  |  |
| 8. Do the figures and tables stand on their own? | V |  |  |  |
| 9. Are the quality control measures documented? | V |  |  |  |
| Quality Rating (Good, Fair or Poor) (See guidance) | 8 (Good) | | | |
| Rater #1 initials: | Du | | | |
| Rater #2 initials: | Zhu | | | |
| *Additional Comments (If POOR, please state why):*  *3. The fMRI images are resampled to voxel size 3x3x3mm, while they were obtained with a 4-mm slice thickness* | | | | |
| *CD, cannot determine: NA, not applicable: NR, not reported | | | | |

| Supplementary Table S6 Li et al. 2022 | + | +/- | - | Other (CD, NR, NA)* |
| --- | --- | --- | --- | --- |
| 1. Did they give a full description of the study participants? | V |  |  |  |
| 2. Did they give a full description of the psychological task used in fMRI? | V |  |  |  |
| 3. Did they specify the spatial normalization procedure, including the atlas or template which is used to match the images to? | V |  |  |  |
| 4. Did they specify how the regions of interest were determined? | V |  |  |  |
| 5. Did they provide enough detail to reproduce the analysis? | V |  |  |  |
| 6. Are all the empirical claims supported by a specific statistical test? | V |  |  |  |
| 7. Did they describe and account for the multiple testing problem? | V |  |  |  |
| 8. Do the figures and tables stand on their own? | V |  |  |  |
| 9. Are the quality control measures documented? | V |  |  |  |
| Quality Rating (Good, Fair or Poor) (See guidance) | 9 (Good) | | | |
| Rater #1 initials: | Du | | | |
| Rater #2 initials: | Zhu | | | |
| *Additional Comments (If POOR, please state why):* | | | | |
| *CD, cannot determine: NA, not applicable: NR, not reported | | | | |

| Supplementary Table S7 Chen et al. 2023 | + | +/- | - | Other (CD, NR, NA)* |
| --- | --- | --- | --- | --- |
| 1. Did they give a full description of the study participants? | V |  |  |  |
| 2. Did they give a full description of the psychological task used in fMRI? | V |  |  |  |
| 3. Did they specify the spatial normalization procedure, including the atlas or template which is used to match the images to? | V |  |  |  |
| 4. Did they specify how the regions of interest were determined? | V |  |  |  |
| 5. Did they provide enough detail to reproduce the analysis? | V |  |  |  |
| 6. Are all the empirical claims supported by a specific statistical test? | V |  |  |  |
| 7. Did they describe and account for the multiple testing problem? | V |  |  |  |
| 8. Do the figures and tables stand on their own? | V |  |  |  |
| 9. Are the quality control measures documented? | V |  |  |  |
| Quality Rating (Good, Fair or Poor) (See guidance) | 9 (Good) | | | |
| Rater #1 initials: | Du | | | |
| Rater #2 initials: | Zhu | | | |
| *Additional Comments (If POOR, please state why):* | | | | |
| *CD, cannot determine: NA, not applicable: NR, not reported | | | | |

| Supplementary Table S8 Li et al. 2023 | + | +/- | - | Other (CD, NR, NA)* |
| --- | --- | --- | --- | --- |
| 1. Did they give a full description of the study participants? | V |  |  |  |
| 2. Did they give a full description of the psychological task used in fMRI? | V |  |  |  |
| 3. Did they specify the spatial normalization procedure, including the atlas or template which is used to match the images to? | V |  |  |  |
| 4. Did they specify how the regions of interest were determined? | V |  |  |  |
| 5. Did they provide enough detail to reproduce the analysis? | V |  |  |  |
| 6. Are all the empirical claims supported by a specific statistical test? | V |  |  |  |
| 7. Did they describe and account for the multiple testing problem? | V |  |  |  |
| 8. Do the figures and tables stand on their own? | V |  |  |  |
| 9. Are the quality control measures documented? | V |  |  |  |
| Quality Rating (Good, Fair or Poor) (See guidance) | 9 (Good) | | | |
| Rater #1 initials: | Du | | | |
| Rater #2 initials: | Zhu | | | |
| *Additional Comments (If POOR, please state why):* | | | | |
| *CD, cannot determine: NA, not applicable: NR, not reported | | | | |

| Supplementary Table S9 Zhe et al. 2023 | + | +/- | - | Other (CD, NR, NA)* |
| --- | --- | --- | --- | --- |
| 1. Did they give a full description of the study participants? | V |  |  |  |
| 2. Did they give a full description of the psychological task used in fMRI? | V |  |  |  |
| 3. Did they specify the spatial normalization procedure, including the atlas or template which is used to match the images to? |  |  | V |  |
| 4. Did they specify how the regions of interest were determined? | V |  |  |  |
| 5. Did they provide enough detail to reproduce the analysis? | V |  |  |  |
| 6. Are all the empirical claims supported by a specific statistical test? | V |  |  |  |
| 7. Did they describe and account for the multiple testing problem? | V |  |  |  |
| 8. Do the figures and tables stand on their own? | V |  |  |  |
| 9. Are the quality control measures documented? |  |  | V |  |
| Quality Rating (Good, Fair or Poor) (See guidance) | 7 (fair) | | | |
| Rater #1 initials: | Du | | | |
| Rater #2 initials: | Zhu | | | |
| *Additional Comments (If POOR, please state why):*  *3. The fMRI images are resampled to voxel size 3x3x3mm, while they were obtained with a 4-mm slice thickness.*  *9. preprocessing lacks removal of linear trend and influence of covariates* | | | | |
| *CD, cannot determine: NA, not applicable: NR, not reported | | | | |

| Supplementary Table S10 Methodological information of studies | | | | | | | | |
| --- | --- | --- | --- | --- | --- | --- | --- | --- |
| Study | **Scanner** | **TR(ms)** | **EO/C** | **Software** | **Threshold** | **Smoothing**  **(FWHM)** | **Coordinate**  **System** | **Quality control for head motion** |
| Wang et al. 2021 | 3T | 2000 | Closed | SPM12 | ***P***<0.05  (corrected) | 6mm | MNI | 1 mm or 1 ° |
| Zhe et al. 2021a | 3T | 2000 | Closed | DPABI | ***P***<0.05  (corrected) | 6mm | MNI | 1.5 mm or 1.5 ° |
| Zhe et al. 2021b | 3T | 2000 | Closed | DPABI | ***P***<0.05  (corrected) | 6mm | MNI | mFD>0.2mm |
| Chen et al. 2022 | 3T | 2000 | Closed | CONN,SPM12 | ***P***<0.01  (corrected) | 6mm | MNI | 3 mm or 3 ° |
| Han et al. 2022 | 3T | 2000 | Closed | DPABI | ***P***<0.05  (corrected) | 8mm | MNI | 2 mm and 1 ° |
| Li et al. 2022 | 3T | 2000 | Closed | DPARSFS, GIFT | ***P***<0.05  (corrected) | 8mm | MNI | 2 mm and 1 ° |
| Chen et al. 2023 | 3T | 2000 | Closed | CONN,SPM12 | ***P***<0.01  (corrected) | 6mm | MNI | 3 mm or 3 ° |
| Li et al. 2023 | 3T | 2000 | Closed | DPARSFS | ***P***<0.01  (corrected) | 8mm | MNI | 1.5 mm or 1.5 ° |
| Zhe et al. 2023 | 3T | 2000 | Closed | DPABI,GIFT | ***P***<0.05  (corrected) | 6mm | MNI | 1.5 mm or 1.5 ° or mFD>0.2mm |
| Abbreviations: *TR, repetition time; EO, eyes open; EC, eyes close; FWHM, full-width at half-maximum; MNI, Montreal Neurological Institute; FD, framewise displacement; SPM, Statistical Parametric Mapping; DPARFS, Data Processing Assistant for Resting-State fMRI; DPABI, Data Processing & Analysis for Brain Imaging; CONN, Functional Connectivity Toolbox;* | | | | | | | | |

| Supplementary Table S11 Results of the jackknife sensitivity analysis | | | | | |
| --- | --- | --- | --- | --- | --- |
| Discarded study | **Precuneus** | **Right middle frontal gyrus** | **Right superior parietal gyrus** | **Left superior temporal gyrus** | **Left midcingulate / paracingulate gyri** |
| Wang et al. 2021 | NO | YES | YES | YES | YES |
| Zhe et al. 2021a | NO | YES | YES | YES | YES |
| Zhe et al. 2021b | YES | YES | YES | YES | YES |
| Chen et al. 2022 | YES | YES | NO | YES | YES |
| Han et al. 2022 | YES | YES | YES | YES | YES |
| Li et al. 2022 | NO | YES | YES | NO | YES |
| Chen et al. 2023 | YES | YES | NO | NO | YES |
| Li et al. 2023 | NO | YES | YES | NO | YES |
| Zhe et al. 2023 | NO | YES | YES | NO | YES |

| Supplementary Table S12 Clusters with increased and decreased activity in patients with vestibular migraine compared with healthy controls in the subgroup of seed-based analysis | | | | | | |
| --- | --- | --- | --- | --- | --- | --- |
| Anatomical region | **Peak MNI coordinate** | | | **SDM-Z** | ***p* value** | **Number of voxels** |
|  | **x** | **y** | **z** |  |  |  |
| VM>HC | | | | | | |
| Right Precuneus | -8 | -72 | 32 | 1.964 | 0.000015497 | 2389 |
| Right middle frontal gyrus | 28 | 28 | 40 | 1.639 | 0.000273526 | 283 |
| VM<HC | | | | | | |
| Left lenticular nucleus, putamen | -32 | -10 | -6 | -2.597 | ~0 | 4275 |
| Left midcingulate / paracingulate gyri | 0 | 6 | 38 | -2.445 | ~0 | 2152 |

**Supplementary Fig S1 Results of funnel plot analysis to test for publication bias**


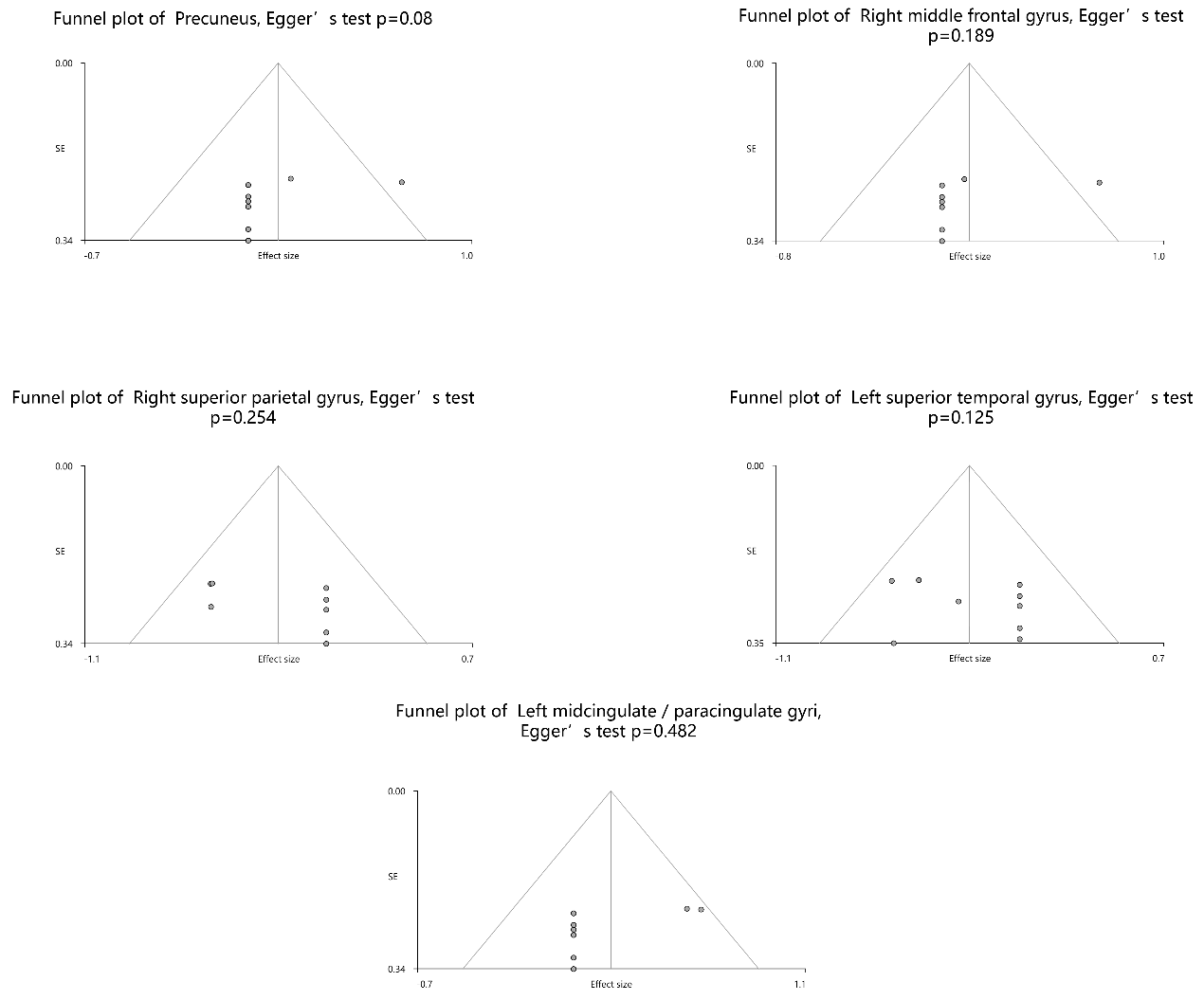

Supplement: Supplementary file 1 — Table S1 Wang et al. 2021. Table S2 Zhe et al. 2021a. Table S3 Zhe et al. 2021b. Table S4 Chen et al. 2022. Table S5 Han et al. 2023. Table S6 Li et al. 2022. Table S7 Chen et al. 2023. Table S8 Li et al. 2023. Table S9 Zhe et al. 2023. Table S10 Methodological information of studies. Table S11 Results of the jackknife sensitivity analysis. Table S12 Clusters with increased and decreased activity in patients with vestibular migraine compared with healthy controls in the subgroup of seed‐based analysis. Figure S1 Results of funnel plot analysis to test for publication bias. [file BRB3-14-e3591-s001.docx]
